# LncRNAs as Chromatin Regulators in Cancer: From Molecular Function to Clinical Potential

**DOI:** 10.3390/cancers11101524

**Published:** 2019-10-10

**Authors:** Rodiola Begolli, Nikos Sideris, Antonis Giakountis

**Affiliations:** 1 Department of Biochemistry and Biotechnology, University of Thessaly, Biopolis, 41500 Larissa, Greece; rbegkolli@uth.gr (R.B.); nsideris@uth.gr (N.S.); 2 B.S.R.C “Alexander Fleming”, 34 Fleming str, 16672 Vari, Greece

**Keywords:** lncRNAs, epigenetics, cancer, chromatin

## Abstract

During the last decade, high-throughput sequencing efforts in the fields of transcriptomics and epigenomics have shed light on the noncoding part of the transcriptome and its potential role in human disease. Regulatory noncoding RNAs are broadly divided into short and long noncoding transcripts. The latter, also known as lncRNAs, are defined as transcripts longer than 200 nucleotides with low or no protein-coding potential. LncRNAs form a diverse group of transcripts that regulate vital cellular functions through interactions with proteins, chromatin, and even RNA itself. Notably, an important regulatory aspect of these RNA species is their association with the epigenetic machinery and the recruitment of its regulatory apparatus to specific loci, resulting in DNA methylation and/or post-translational modifications of histones. Such epigenetic modifications play a pivotal role in maintaining the active or inactive transcriptional state of chromatin and are crucial regulators of normal cellular development and tissue-specific gene expression. Evidently, aberrant expression of lncRNAs that interact with epigenetic modifiers can cause severe epigenetic disruption and is thus is closely associated with altered gene function, cellular dysregulation, and malignant transformation. Here, we survey the latest breakthroughs concerning the role of lncRNAs interacting with the epigenetic machinery in various forms of cancer.

## 1. Introduction

The term epigenetics was first used to describe “the branch of biology which studies the causal interaction between genes and their products which bring the phenotype into being” [1]. Today, epigenetics refers to an amalgamation of modifications that result in changes in chromatin, which collectively regulate gene expression with no change to the underlying DNA sequence [2]. These modifications are present in most organisms and their effects can be inherited under certain circumstances [3,4]. Epigenetic alterations are broadly divided into DNA and histone modifications. The first category refers to the addition of 5’-methyl groups to cytosines. These cytosines are not randomly distributed in the genome but are organized in stretches of CG dinucleotides, known as CpG islands. The presence of methylated cytosines in promoter and/or enhancer regions is broadly thought to prevent binding of transcription factors, ultimately repressing gene expression. The second category comprises post-translational modifications of histone tails such as, but not limited to, addition or removal of methyl, acetyl, and phospho groups to and from the N-terminal ends of histone amino acid residues [5,6,7]. Such modifications affect nucleosome charge, thereby altering chromatin structure and subsequently conferring a transcriptionally active (euchromatic) or inactive (heterochromatic) state through the recruitment of transcriptional co-activators [8,9]. For example, histone methylation marks such as H_3_K_4_me_2_, H_3_K_4_me_3_, H_3_K_9_me_1_, and acetylation in the form of H_3_K_27_ac are frequently associated with open chromatin and enhancer/promoter activation, while H_3_K_27_me_2_, H_3_K_27_me_3_, H_3_K_9_me_2,_ and H_3_K_9_me_3_ are associated with inactive chromatin state, resulting in gene repression [10,11,12,13,14]. Histone modifications are mediated by an assortment of enzymes such as DNA methyltransferases and histone methyl/acetyltransferases (and their modification removal counterparts), collectively known as the epigenetic machinery. Well-known representatives in mammals include the DNA Mehtyltransferase (DNMT) family, which facilitates DNA methylation, the Polycomb Respressive Complex 2 (PRC2) and its catalytic component Enhancer of Zeste Homolog 2 (EZH2), which promote H_3_K_27_ methylation, and the Lysine Acetyltransferase (KAT) and Histone Deacetylase (HDAC) families that mediate histone acetylation and deacetylation, respectively [2,14,15,16]. A notable example of epigenetic regulation with implications in physiology includes the process of gene imprinting and X chromosome inactivation through methylation of CpG islands [17,18].

Deregulation of the “normal” epigenetic context can lead to aberrant gene activation or repression that is closely linked with various human pathologies, most notably cancer [19,20,21]. Cancer itself is a heterogenous disease, the molecular basis of which has been attributed to both intrinsic (e.g., genetic and epigenetic) as well as extrinsic (environmental) factors. A major driving force of carcinogenesis is the accumulation of mutational burden in malignant cell populations that disrupts key signaling networks responsible for homeostasis, development, and differentiation, ultimately leading to cancer cell immortalization, expansion, and distant organ metastasis [22,23,24]. In addition, aberrant epigenetic modifications can lead to nonphysiological function of *cis/trans* regulatory elements, ultimately resulting in overexpression of oncogenes and/or silencing of tumor suppressors [25,26,27]. Technical advancements in deep sequencing technologies, giving rise to the field of cancer epigenomics, have been utilized in order to map and contrast epigenetic modifications between normal and tumor tissues [28,29,30,31].

DNA methylation is the most thoroughly characterized epigenetic modification [32,33]. Most cancer types seem to exhibit a genome-wide hypomethylation signature compared with normal adult tissues, leading to ectopic activation of physiologically silent oncogenes. Moreover, DNA hypomethylation is often paired with re-animation of transposable elements, leading to genomic instability and chromosomal rearrangements, both of which are well-established molecular hallmarks of most cancer subtypes [34,35,36]. In sharp contrast to the global hypomethylation signature, most tumors exhibit patterns of localized promoter hypermethylation of CpG islands, leading to epigenetic silencing of tumor suppressors and subsequent expansion of tumor cell subpopulations [19,37]. Finally, mutations in histone-modifying enzymes, such as the previously mentioned EZH2 can elicit protein hyperactivity or inactivity, leading to condensation or relaxation of chromatin loci that contributes further to ectopic gene expression and poor patient outcome [38,39,40].

Thorough characterization of the human transcriptome led to the discovery of a novel class of noncoding transcripts, named long noncoding RNAs (lncRNAs) [41]. These RNA species are typically longer than 200 nt, exhibit low or no protein-coding potential, and function primarily as regulators of gene expression. Their biogenesis and fundamental properties mirror those of protein-coding genes, since lncRNAs are typically transcribed by RNA pol-II, possess a 5’ methyl-cytosine cap and 3’ poly-A tail, and often exhibit alternative splicing patterns [42]. Main differences compared with typical protein-coding genes, and apart from the negligible coding potential of lncRNAs, are their poorer conservation (at least in terms of primary sequence) between evolutionary taxa, their overall low levels of expression, as well as the fact that lncRNAs exert their regulatory functions through their tertiary structures [41,42,43,44,45]. LncRNAs are expressed in most tissues (stem cells, epithelial cells, endothelial cells, tumor cells, etc.) and demonstrate high tissue- and/or cell-specific patterns of expression [46,47]. LncRNAs have also been shown to regulate a variety of cellular functions such as (post)transcriptional activity, chromatin remodeling, and protein interactions in both the nucleus and the cytoplasm, ultimately orchestrating processes such as cellular division and development [41,48,49,50]. A very common cytoplasmic function is miRNA sponging, where lncRNAs function as molecular decoys to protect mRNA targets from miRNA-mediated inhibition. Inside the nucleus, lncRNAs have been shown to interact with transcription factors and epigenetic modifiers, acting as guides, scaffolds, or stabilizers that alter chromatin structure and gene expression [51,52]. One of the best-studied interactions of lncRNAs with the epigenetic machinery is provided by Xist, which mediates X chromosome inactivation via interaction with and guidance of histone methyltransferases [53,54]. A large number of studies have highlighted the involvement of the noncoding transcriptome in establishing cancer epigenetic activities, either through direct physical interactions with epigenetic modifiers, or through regulation of their expression, stability, and post-translational modifications (Table 1) [55,56,57,58].

It is evident that dysregulation of lncRNAs can result in aberrant gene expression, a state that is intimately linked with malignant cellular transformation. Due to the specificity of their expression patterns, lncRNAs are ideal diagnostic biomarkers. Their elaborate tertiary structures can provide the basis for developing novel pharmaceutical approaches. In this review, we are focusing on the latest breakthroughs concerning the interactions of lncRNAs with the epigenetic machinery, we present examples of their involvement in various forms of cancer epigenetics and explore their diagnostic and therapeutic potential.

## 2. Molecular Functions of lncRNAs as Regulators of Chromatin

The molecular function of several lncRNAs is mediated through interactions with the chromatin. Such lncRNA–chromatin interactions can be subdivided into two types based on whether they alter three-dimensional chromatin architecture. The first type includes lncRNA–chromatin interactions that are restricted to physically separated target loci and regulate gene expression through histone and/or DNA modifications without affecting three-dimensional architecture. The second type refers to lncRNAs that overcome physical separation of target regulatory elements and regulate gene expression by initiating and/or maintaining chromatin loops between enhancers and promoters on a three-dimensional space. Below, we highlight different examples of lncRNA-mediated regulation of chromatin, starting with examples of lncRNAs that affect histone methylation.

### 2.1. lncRNAs as Regulators of Histone Methylation

**Figure 1:**
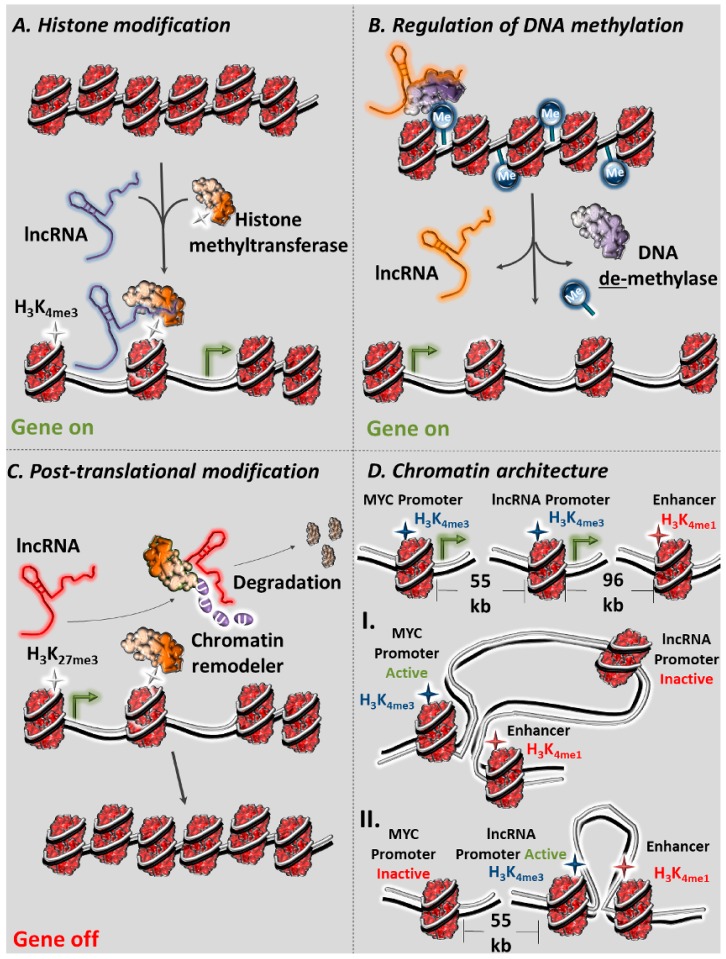
Molecular function of lncRNAs in chromatin regulation. (**A**) lncRNAs, such as HOTAIR, interact with chromatin remodelers such as histone methyltransferases in order to induce or suppress histone tail methylation and subsequently gene expression. (**B**) Other lncRNAs, such as TARID, regulate gene expression through demethylation of target genes. (**C**) Another implication of lncRNA function in chromatin regulation includes ANCR that regulates the post-translational stability of chromatin remodelers. (**D**) Apart from the transcript itself, regulatory elements of lncRNAs, such as the promoter of PVT1, compete with promoters of gene for interaction with common enhancers, indirectly regulating the latter in cancer.

Well-established examples of lncRNAs that dictate histone methylation in cancer comprise lncRNAs HOTAIR, HOTTIP, and ANRIL. HOTAIR, which is transcribed from the HOXC locus, has been shown to interact with PRC2 to facilitate epigenetic silencing of the HOXD locus by establishing H_3_K_27_me_3_ [78]. ANRIL, which is transcribed in an antisense orientation to the INK4 locus, also binds to the SUZ12 subunit of PRC2 and mediates H_3_K_27_me_3_ epigenetic silencing of the CDKN2A/B locus [79]. HOTTIP, another lncRNA transcribed from the HOXA locus, interacts with the WDR5/MLL histone methyltransferase complex and mediates gene activation of HOXA in cis via H_3_K_4_me_3_ alterations [80]. NEAT1 has been known for its essential role in maintaining the structural integrity of paraspeckles under normal conditions [81]. Paraspeckles are subnuclear RNA–protein structures that are critical regulators of gene expression through nuclear retention of target RNAs [82]. At the molecular level, NEAT1 interacts specifically with H_3_ and establishes H_3_K_4_me_3_ and H_3_K_9_ac open chromatin marks in promoter regions of its targets (Figure 1A). Data from ChIRP-seq (Chromatin Isolation by RNA Purification followed by sequencing, a modification of ChIP in which biotinylated oligos that are complementary to a RNA of interest are used in order to pull down its interacting chromatin) support NEAT1’s high affinity recruitment to, among others, the prostate-specific membrane antigen (PSMA) promoter, facilitating its epigenetic activation by guiding members of the epigenetic machinery [59,83].

LncRNA MEG3 is an interesting example of the crosstalk between lncRNAs and epigenetics. In 2015, a thorough investigation of the ability of MEG3 to interact with chromatin in breast cancer cells was conducted. The research group screened for repressive chromatin-associated lncRNAs by comparing EZH2 and H_3_K_27_me_3_ ChRIP-seq data (Chromatin RNA immunoprecipitation followed by high-throughput sequencing, a modification of RIP in which an antibody is used to immunoprecipitate chromatin-interacting RNA after inhibition of transcription) and identifying MEG3 among their hits. At the molecular level, MEG3 lncRNA exerts its regulatory function through a GA-rich motif, which forms RNA–DNA triplex structures with abundant GA sequences in the distal regulatory elements of the target genes. Through a combination of ChOP (Chromatin Oligo affinity Purification, a modification of ChIRP-seq, presented above), ChIP-seq, and 3C assays, it was demonstrated that MEG3/ H_3_K_4_me_1_-bound regions interacted with the TGFBR1 promoter, pointing to the formation of an R loop between distal regulatory elements and the target promoter [61].

Other examples demonstrate the functional flexibility of lncRNAs that commonly exhibit multiple regulatory roles across diverse cellular processes [84,85]. TUG1 is a notable example of this category, functioning in both the nucleus and cytoplasm. In the nucleus, TUG1 acts a molecular scaffold that bridges PRC2 to the YY1 transcription factor, guiding the newly formed complex to downstream target genes involved in neuronal differentiation including, but not limited to, *BDNF*, *NGF*, and *NTF3*. Localization of the complex to target promoter regions elicits epigenetic silencing through establishment of the repressive H_3_K_27_me_3_ mark that ultimately promotes tumor aggressiveness. It is proposed that YY1 acts as a form of chromatin-specific guide to target gene loci. The fact that the TUG1 sequences responsible for interaction between TUG1, PRC2, and YY1 have been identified and seem well conserved between humans and mice, marks this transcript as a therapeutic candidate of self-renewal in Glioma Stem Cells (GSC). In the cytoplasm, TUG1 acts as a miRNA sponge for miR-145. Sponging of this miRNA, protects SOX2 and MYC transcripts from subsequent degradation [60].

### 2.2. lncRNAs as Regulators of Histone Acetylation

Apart from histone methylation, other lncRNAs regulate gene expression through histone acetylation [86,87]. One example of an lncRNA with a crucial role in regulating the process of cell differentiation through its interaction with the SIRT6 deacetylase is lncPRESS1. SIRT6 de-acetylates the H_3_K_56_/K_9_ac active chromatin-associated mark and represses a repressive state to target chromatin regions [88]. Essentially, lncPRESS1 acts as a molecular decoy for SIRT6, preventing targeting of the chromatin of genes that controls the transition from the pluripotent to differentiated state in ESCs(Embryonic Stem Cells) from SIRT6. Collectively, it was shown that this transcript has a pluripotency-specific signature and its presence assists in maintaining ESC pluripotency. Expression of lncPRESS1 seems to be under the control of p53, which recruits histone modifiers to the lncPRESS1 locus and establishes repressive chromatin marks [73]. Interestingly even though this transcript has not yet been directly associated with a form of cancer, its function as a pluripotency regulator in ESCs raises the possibility of a role also in cancer stem cells. 

### 2.3. lncRNAs as Regulators of DNA Methylation

As described earlier, DNA methylation plays a crucial role in gene expression regulation and chromatin architecture and has a key role in malignant transformation when aberrant methylation patterns emerge [89]. Methylation is usually present in CpG islands in promoter regions and is associated with the inactive chromatin state, while on the other hand, demethylation of those regions confers activity and allows binding of transcription factors [90]. TARID is a long intergenic transcript whose promoter region is located within the third CpG island of the TCF21 gene and is expressed antisense to TCF21. TARID binds GADD45A, a DNA repair protein that promotes active demethylation, and guides it along with its interactor protein TDG to the TCF21 promoter (Figure 1B) [91]. This colocalization is responsible for demethylation of the TCF21 promoter, and its subsequent activation mediates hydroxy-methylation of CpG residues through association with the TET protein family, a key intermediate step important in head and neck squamous cell carcinoma (HNSCC) [92]. 

Recent data demonstrate that TARID in fact guides GADD45A to the TCF21 promoter and forms an R-loop with the promoter, which is recognized by the GADD45A protein as a region marked for demethylation. GADD45A in turn recruits the TDG and TET proteins required for demethylation [68]. As TCF21 is a transcription factor with tumor-suppressing properties that is epigenetically silenced in cancer, its activation by TARID could result in suppression of aberrant characteristics. Notably, formation of functional R-loops seems to be a widespread mechanism for lncRNAs to interact with gene loci, with new cases being discovered, and their occurrence not limited to cancer. Another example includes the convergent transcription of an antisense lncRNA in the Pcdh gene cluster which facilitates regulatory interactions within a gene cluster in a stochastic manner during neuronal differentiation [93].

### 2.4. lncRNAs as Post-Translational Regulators of the Epigenetic Apparatus

The interplay between lncRNAs and the epigenetic apparatus is not limited to recruitment and/or scaffolding functions that result in chromatin remodeling [18]. LncRNAs, have been identified that are capable of regulating protein complex stability by promoting or inhibiting protein degradation [72,94,95,96]. LncRNA ANCR is an interesting example of this type. ANCR has been shown to bind directly to EZH2, facilitating its degradation (Figure 1C). More to the point, ANCR is necessary for the binding of CDK1 to EZH2, where CDK1 marks EZH2 for ubiquitin-proteasome degradation via Thr-345 and Thr-487 phosphorylation in breast cancer cells. In breast cancer, where ANCR remains transcriptionally dormant, hyperactivity of EZH2 leads to an increase of the repressive H_3_K_27_me_3_ mark in promoter regions of EZH2 target genes (such as tumor-suppressors E-cadherin, HOXA10, etc.) which normally repress EMT progression [69].

LUCAT1 is another example of an lncRNA that post-translationally regulates another chromatin remodeler, this time DNMT1, which controls DNA methylation. Downregulation of LUCAT1 has been linked to lower protein levels of DNMT1 and subsequent reduction of repressive methylation marks in known downstream tumor-suppressor genes under the control of DNMT1. LUCAT1 inactivity has also been correlated with higher protein levels of UHRF1, a protein shown to induce the ubiquitination of DNMT1 [71]. This evidence, along with the fact that LUCAT1 physically binds to DNMT1 suggests that LUCAT1 functions as a stabilizer for the latter, staving off its degradation [70].

### 2.5. lncRNAs as Modifiers of Chromatin Three-Dimensional (3D) Architecture

Enhancers are key regulatory elements which allow for the binding of transcription factors, leading to transcriptional activation of downstream genes. Such transcriptional activation requires interaction of enhancer elements with gene promoters [97]. Enhancers regulate their gene targets over vast genomic distances, with a number of proteins such as cohesins along with various RNA intermediates facilitating local 3D chromatin rearrangements in order to bridge the two regulatory elements [98,99]. The involvement of lncRNAs in chromatin looping and enhancer–promoter interactions has been observed on many occasions, often with implications in human disease [100,101]. Additional evidence supporting the involvement of lncRNAs in transcriptional regulation via modulation of 3D chromatin architecture is their association with RNA-binding proteins (RBPs) in the nucleus [102,103]. RBPs seem to localize in active chromatin, especially in gene-promoter regions and appear, in some cases, to be directly involved in the processes of transcriptional control. Some RBPs have been shown to interact with DNA in an RNA-dependent manner, while others have the capacity to act as transcription factors that modify chromatin architecture. A notable recent example involves the association of the RBM25 RBP with the RNA-dependent transcription factor YY1, which facilitates chromatin binding, DNA looping and transcription in HepG2 and K562 human cell lines [104]. lncRNAs such as Xist, Airn, and Kcnq1ot1 have been shown to induce deposition of inactive chromatin marks over large genomic distances with the help of RBPs [105,106]. Selected examples of lncRNAs that modify chromatin architecture through various mechanisms are highlighted below.

A recent study identified several complex trait/disease-associated intergenic lncRNAs (lincRNAs) in human lymphoblastoid cells. It was observed that these loci are usually localized at TADs (Topologically Associated Domains), which are often packed with enhancer-like elements. DNA–DNA interactions and abundant cohesion-binding sites in TAD regions are commonplace [107]. Regulation of neighboring protein-coding genes by TAD lncRNAs in their vicinity, along with the location of enhancer-rich TAD signatures suggests these noncoding transcripts play a role in influencing local chromatin architecture. Interestingly, TR-lincRNAs are conserved in humans and have been shown to interact with disease-relevant loci, including loci associated with various forms of cancer [108].

Involvement of lncRNAs on chromatin remodeling is not always facilitated by the lncRNA transcript itself, but sometimes the act of lncRNA transcription has a functional role. A notable example is lncRNA ThymoD, which determines T-cell differentiation through the regulation of the Bcl11b gene. Transcription of ThymoD has been linked to demethylation of CpGs, associated with CTCF-binding sites and the recruitment of the cohesin complex, promoting the deposition of active epigenetic marks and the H3.3 histone variant mark across the Bcl11b intergenic domain. This in turn leads to repositioning of a distant Bcl11b-enhancer element from the nuclear lamina (inactive chromatin site), via chromatin looping, to the Bcl11b promoter (active chromatin site) [74].

Apart from the lncRNA transcripts, their regulatory elements (e.g., lncRNA promoters) can individually function as autonomous regulators of distant loci (Figure 1D). A characteristic example is the role of the PVT1 lncRNA promoter that limits MYC expression in breast cancer, essentially acting as a tumor-suppressor element. In breast cancer cells, a combination of 4C-seq (modification of chromosome conformation capture, a method that combines cross-linking, circulating ligation, and deep sequencing in order to detect interactions between a single DNA locus with the rest of its associated chromosome), ATAC-seq (assay for transposable accessible chromatin followed by high-throughput sequencing, a method that relies on Tn5 transposase and deep sequencing in order to map genome-wide chromatin accessibility), and HiChIP (modification of ChIP in which an antibody is used in order to immunoprecipitate three-dimensional chromatin interactions that are bound by the target protein) experiments proved that PVT1 promoter competes with the promoter of MYC for interaction with a set of enhancers [109,110,111]. Interestingly, CRISPRi (CRISPR-inhibition) targeting PVT1 promoter led to enhancement of MYC expression and cancer cell proliferation, while reversal of the interference abolished MYC activity. Hybrid mouse experiments showed that PVT1 promoter was only capable of regulating MYC in cis [75]. The PVT1 promoter is a target of genetic mutations and chromosomal rearrangements in various forms of cancer, further highlighting its regulatory importance [112]. In conclusion, lncRNAs function as epigenetic regulators of gene expression through a variety of different mechanisms.

## 3. Epigenetically Regulated lncRNAs

Solid tissues consist of a heterogenous mixture of different cell types including epithelial, vascular, blood, lymphatic, and immune cells, which altogether make up the tumor microenvironment [113]. These cellular elements specialize gene expression through epigenetic processes such as histone deacetylation, which in turn creates an intricate signaling network of cell-to-cell communication, allowing the tumor to thrive. Notably tumors have been known to take advantage of immune cells and inflammatory signaling networks so as to avoid induced apoptosis in a process known as immune evasion [114,115]. Recently, a connection was made between this phenomenon and the regulatory role of NKILA, an NF-κB interacting lncRNA. Although this lncRNA does not exert its regulatory function through the epigenetic machinery, it is itself an important example of an epigenetically regulated transcript. Downregulation of NF-κΒ signaling is associated with activated induced cell death (AICD) in cytotoxic T lymphocytes (CTLs), one of the organism’s major defense systems against cancer cells [116]. NKILA’s role in the pathway starts with antigen recognition by CTLs, which triggers calcium influx, leading to a large-scale nuclear translocation of calmodulin, a calcium-binding protein. Nuclear calmodulin interacts with HDACs and inhibits deacetylation of the NKILA promoter, contributing to a more active chromatin state through maintenance of H_4_ac, H_3_K_27_ac, and H_3_K_9_ac marks, which in turn facilitate binding of STAT1 [66].

Another example of an epigenetically regulated lncRNA is TP53TG1. In normal cells, this lncRNA interacts with the YBX1 RNA-binding protein, preventing its translocation from the cytoplasm to the nucleus. In TP53TG1 nonexpressing tumors, YBX1 accumulates in the nucleus and subsequently activates the PI3K/AKT pathway, resulting in degradation of the p53 tumor-suppressor protein [67]. 

## 4. LncRNAs as Diagnostic and Therapeutic Targets in Cancer 

During the last decade, significant progress in the field of genomics, coupled to the joined efforts of international consortia such as The Cancer Genome Atlas (TCGA) and the International Cancer Genome Consortium (ICGC), collectively integrated genomic and clinical information for thousands of cancer biopsies [30,117]. The outcome of these multidimensional analyses was the creation of publicly accessible databases that link genetic, epigenetic, and transcriptional abnormalities to disease progression with unprecedented detail. Such in-depth characterization of the cancer genome revealed several lncRNAs that are functionally involved in initiation, evolution, and spread of tumor cells. Several lncRNAs that participate in the epigenetic regulation of gene expression also have a role in disease progression, providing novel diagnostic and therapeutic opportunities as well as challenges.

### 4.1. Clinical Impact of lncRNAs Involved in Regulation of Chromatin

**Figure 2:**
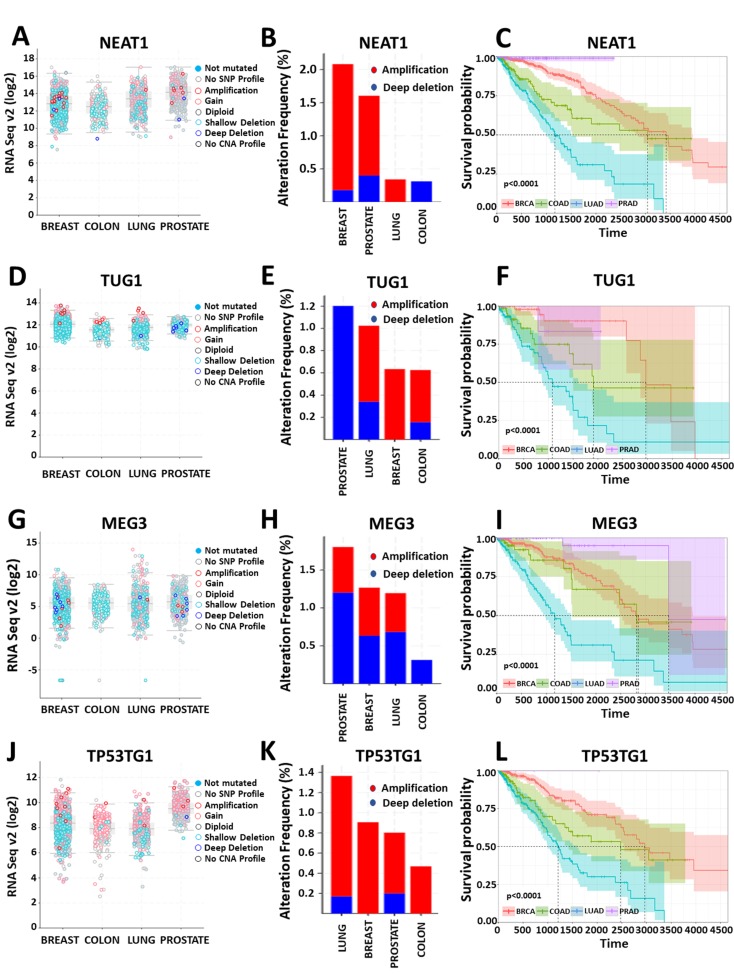
Expression, genetic alteration, and survival analysis of selected lncRNA epigenetic regulators in the most frequent types of cancer based on TCGA (The Cancer Genome Atlas) data. Left panels (**A,D,G,J**) correlate lncRNA gene expression with copy number variation across cancer types. Middle panels (**B,E,H,K**) demonstrate amplification and/or deep deletion percentage of each lncRNA gene among patients for each cancer type. Right panels (**C,F,I,L**) demonstrate average impact on survival time for each cancer type, based on intermediate lncRNA expression (patient stratification cut-offs: >Q1 and <Q3). Dashed lines in Kaplan–Meier plots represent median survival time, *p*-values correspond to statistical significance of survival difference between all tumor types for each lncRNA. (**A–C**) Expression, genetic amplification/loss, and survival analysis of NEAT1. Gene copies of this lncRNA are increased in breast and prostate compared with the other cancer types. (**D–F**) Expression, genetic amplification/loss, and survival analysis of TUG1. This lncRNA has minimum expression variation between cancer types. (**G–I**) Expression, genetic amplification/loss, and survival analysis of MEG3. This lncRNA has elevated expression in lung cancer patients. (**J–L**) Expression, genetic amplification/loss, and survival analysis of TP53TG1. This lncRNA is amplified in most cancer types and shows elevated levels of expression in prostate cancer. All data are reanalyzed from UCSC (University of Santa Cruz California) Xena.

HOTAIR is a notable example of chromatin-regulating lncRNAs that function as oncogenes. Elevated expression of this lncRNA has been associated with disease progression, invasion, and metastasis for a variety of cancer types [118,119]. HOTTIP, another oncogenic lncRNA with chromatin-regulating properties, has been linked to pancreatic cancer growth and metastasis [63,120]. NEAT1, which is highly expressed in aggressive prostate tumors, partially due to genetic amplification (Figure 2A–C), has been positively correlated with therapeutic resistance in cancer. Overexpression of this lncRNA promotes proliferation and invasion of prostate cancer cells, while knockdown of its expression suppresses them [59]. Interestingly, apart from its oncogenic role in prostate cancer, this lncRNA can also function as a tumor suppressor in other cancer types [121]. ANRIL is an example of a noncoding locus that is targeted by mutations that predispose for various cancer types [122,123]. Elevated levels of ANRIL are associated with acceleration of disease progression and shortening of survival time [124,125]. Moreover, increased expression of TUG1 (Figure 2D–F) in the cytoplasm and the nucleus collectively promotes poor disease outcome for a variety of cancer types [126,127]. LUCAT, another lncRNA that is upregulated in cancer, contributes to disease progression in esophageal squamous cell carcinoma (ESCC) by promoting proliferation and migration of tumor cells [70]. Upregulation of the lncRNA NKILA in cytotoxic T lymphocytes of breast and lung cancer patients is one of the determining factors of activated induced cell death sensitivity that in turn has been correlated with poor survival [66]. Finally, with regards to the ThymoD’s function in chromatin architecture of T-cells, considering that T-cell fate is under stringent transcriptional regulation and that absence of ThymoD expression has been linked with various malignancies, detailed investigation into this form of regulation is needed for development of possible treatments and diagnosis.

In sharp contrast, lncRNAs with tumor-suppressing function, such as MEG3 (Figure 2G–I) are downregulated in several cancer types [61,128,129]. Another such example is ANCR, elevated activity of which has been shown to suppress migration and invasion of breast cancer cells both in vitro and in vivo. Finally, TP53TG1 (Figure 2J–L) is a tumor suppressor that is actively transcribed in response to DNA damage and p53 induction. It is frequently deleted in breast tumors and silenced in gastric and colorectal tumors due to cancer-specific CpG island hypermethylation in its promoter. Inactivation of TP53TG1 has been shown to confer enhanced resistance to generic chemotherapy in gastrointestinal tumors, while its promoter hypermethylation has been linked with poor disease outcome [67]. 

### 4.2. Diagnostic Potential of lncRNAs in Cancer 

Τhe deregulation and functional involvement lncRNAs in cancer provides novel opportunities for expanding the existing diagnostic and therapeutic toolbox of this complex disease. With regards to diagnosis, the discovery of circulating oncogenic lncRNAs in tumor-derived exosomes coupled to their specific spatiotemporal activation currently holds great promise towards the development of highly specific diagnostic markers [130,131]. Exosomes are a group of extracellular vesicles that arise when intermediate endosomal compartments, known as multivesicular bodies (MVBs), fuse with the plasma membrane so as to release their contents [132,133]. Exosomes appear to function as vehicles of cell-to-cell communication and have been implicated in various diseases, including cancer [132,134]. These vesicles range from sizes of 30–100 nm and engulf a wide assortment of molecular cargos such as proteins, lipids, and nucleic acids including mi/mRNAs as well as lncRNAs [135,136]. Several lncRNAs that epigenetically regulate cancer cells through various mechanisms are also part of the exosomal cargo that is secreted from tumors. 

Examples of lncRNAs that interact with the epigenetic machinery and have been detected in exosomes are MEG3 and HOTAIR that are secreted specifically from cervical tumors, but not from their normal counterparts, offering an opportunity for developing RNA-centric diagnostic approaches [137]. Other examples of lncRNAs that are secreted from tumor exosomes include LUCAT1 and PVT1 in exosomes of liver cancer [138,139]. In sharp contrast to lncRNA tumor-specific presence in extracellular vesicles, secreted exosomes from normal intestine carry significantly higher levels of HOTTIP than their colon cancer counterparts, providing novel opportunities for monitoring disease onset [140]. Interestingly, exosomal packaging appears to increase stability (and therefore detection threshold) of NEAT1 and certain other lncRNAs compared with their intracellular levels [141].

Evidence suggests that apart from being secreted, lncRNAs can themselves exert an important level of control on the very production of exosomes in cancer. On that note, lncRNA-APC1, which is downregulated in colorectal carcinoma cells (CRCs), due to mutations of its master regulator APC, is a tumor-suppressor transcript which inhibits angiogenesis, proliferation, and migration of cancer cells. With exosomes playing a vital role in the induction of angiogenesis in CRCs, it has been shown that LncRNA-APC1 exerts its function by decreasing the stability of Rab5b mRNA, an important regulator of the exosome production process, ultimately reducing overall exosome production [142]. 

### 4.3. Therapeutic Potential of lncRNAs in Disease

Complementary to their role in diagnosis and prognosis, lncRNAs possess multiple features that highlight them as promising therapeutic agents for human diseases. Their unique expression patterns, distinguished by high tissue or cell type specificity, render them as excellent markers for high-precision targeting of cancer subtypes. This, coupled with their generally low levels of expression, compared with those of their coding counterparts, provide the basis for efficient knockdown therapeutic approaches [143,144]. Free of coding restrains, their sequence accumulates and maintains aberrant mutations that frequently predispose directly or indirectly (as part of haplotypes) to cancer, allowing for early prognosis of hereditary forms of cancer [145,146]. Considering the above, it is no surprise that several biotechnological companies such as OPKO-CURNA, RaNA and TransSINE orient their therapeutic efforts towards lncRNAs.

Classical lncRNA therapeutics in cancer involve the use of nucleic acid-based knockdown techniques. Examples include successful in vivo RNAi targeting of lncRNAs such as PANDA, MALAT1, and HOTAIR [62,147,148]. A similar approach utilizes DNA oligonucleotides (antisense oligonucleotides, ASOs) which hybridize with the target leading to RNase-H mediated degradation, as demonstrated in vivo for BDNF-AS and MALAT1 [149,150]. Similarly, nonionic DNA analogs, named morpholinos, which appear capable of binding to RNA and promoting its degradation, have entered preclinical trials [151]. Additional undergoing preclinical studies include aptamers, which are oligonucleotides that target small molecules through the formation of efficient complementary 3D shapes, and (deoxy-) ribozymes which bind to specific targets and independently catalyze their cleavage [42,152,153].

Recent advancements in synthetic biology have given rise to new RNA therapeutic strategies by taking advantage of artificial genetic circuitries. A novel approach focuses on manipulating human immunology to produce modified T-cells designed to be more effective against cancer cells [154]. Interventions in immune and inflammatory signaling of immune cells within the tumor environment can be achieved through the use of RNA aptamers which mimic or block signaling molecules such as interleukins [155]. Α developing approach involves the use of the CRISPR/Cas9 system to reactivate or silence tumor-suppressing or oncogenic lncRNAs respectively [156,157]. In a very intriguing variation of this system, the sgRNA is coupled with a signal-receiving aptamer, forming an artificial circuit that is capable of regulating and even rewiring intracellular signaling in response to specific signals [158].

Perhaps the most promising therapeutic aspect of lncRNAs lies within their regulatory interplay with protein complexes, such as the epigenetic apparatus, facilitated through their complex RNA tertiary structure. Small and easily diffused antagonists that bind to lncRNA stem-loops and prevent them from interacting with their protein counterparts can pave the way towards the development of efficient and highly specific therapeutic agents free of the side-effects that arise from blocking conserved protein receptors [159,160]. Currently, a number of small aptamers, designed to target lncRNAs secondary structures have been engineered and tested as anticancer drugs [161,162]. Screens for small chemical molecules, capable of binding to specific lncRNA sequences and preventing them from acquiring proper folding or from interacting with their protein partners are being performed and are leading to the development of novel pharmaceutics that harness hyperactivation of epigenetic modifiers in order to specifically de-repress tumor-suppressor activity [163,164].

## 5. Challenges and Limitations of lncRNA Biology 

Undoubtedly, with their discovery lncRNAs shed light to a new regulatory layer of complex disease, providing opportunities for expanding existing diagnostic and therapeutic approaches. However, lncRNA biology also comes with a variety of challenges and limitations, which also apply to lncRNAs that function through the regulation of cancer chromatin. Free access to genomics data promotes the discovery of lncRNAs that are deregulated in tumors, yet only a small portion of them have been mechanistically characterized. Functional dissection of lncRNAs is generally more challenging than the one of their protein-coding counterparts, since lncRNAs often function via complex combinations of RNA–RNA, RNA–protein and RNA–chromatin interactions. This in turn calls for application of an arsenal of demanding molecular approaches [159,165]. Another limitation rises from the generally low levels of expression that obstruct easy detection and again require use of more advanced methods [166]. Moreover, in terms of primary sequence, human lncRNAs are poorly conserved in mice, raising difficulties for in vivo evaluation of their mechanistic and/or clinical role in well-established biomedical murine models. In such cases, deployment of xenografts derived from patients or human cell lines provides an alternative for understanding how the relevant lncRNA operates within the tumor microenvironment [62,167].

At the clinical level, such limitations demand careful design of lncRNA-centered therapeutics. Researchers need to fully understand the whole functional spectrum of a given lncRNA in order to a priori predict and evaluate possible side effects of its therapeutic exploitation. Another complication stems from the multifunction of lncRNAs. As disease progresses, the same lncRNA may interact with multiple molecules in order to regulate different subgroups of genes between tissues or cell subpopulations [168]. Consequently, lncRNAs such as NEAT1 (see above) can act as both tumor-suppressors and oncogenes depending on the malignant tissue and disease stage. Despite these limitations, ongoing research efforts with clinical applications focus on lncRNAs with the aim of evaluating better the full potential of RNA-centric approaches for the patient [169].

## 6. Conclusions

Following the advance of the last decade in the fields of cancer epigenomics and transcriptomics, an increasing interest in lncRNAs that mechanistically interact with the epigenetic machinery and facilitate tumorigenic chromatin remodeling has been developed. These noncoding epigenetic regulators act as a driving force for promoting or suppressing cancer progression. Such lncRNAs also have the potential to act as novel diagnostic biomarkers as well as therapeutic targets for inhibiting malignant transformation or disease progression. Recently, it has been shown that lncRNAs are not operationally restricted to their original cellular environment but can be packed inside exosomes, secreted from cancer cells and subsequently act as paracrine signaling effectors at distant tissues. Identification of circulating tumor-derived exosomal lncRNAs could also be utilized in precision diagnostics. Given that lncRNAs exert much of their regulatory control through interactions of their complex tertiary structures with protein complexes and epigenetic regulators, several approaches that inhibit proper RNA folding or RNA–protein interactions are being explored as modern cancer therapeutics.

## Figures and Tables

**Table 1:** Examples of mechanisms through which lncRNAs are involved in cancer chromatin regulation [59,60,61,62,63,64,65,66,67,68,69,70,71,72,73,74,75,76,77].

Mechanistic Classification	LncRNA	Cancer/Cell Type	Mechanism	Interactor	Target	Functional Impact	Ref
**Histone methylation**	NEAT1	Prostate	Facilitates H_3_K_4_me_3_ and H_3_K_9_ac	Unknown	PSMA promoter	Cell proliferation and invasion	[59]
	TUG1	Glioma	Epigenetic transcriptional silencing via H_3_K_27_me_3_	EZH2, YY1	BDNF, NGF and NTF3	Maintenance of stemness features of Glioma Stem Cells (GSCs) through exon 1	[60]
	MEG3	Breast	Guides PRC2 through RNA-DNA triplex structure	PRC2 (EZH2)	TGF-b pathway genes	Not well defined	[61]
	HOTAIR	Breast	PRC2 genomic relocalization and gene silencing through H_3_K_27_me_3_	PRC2	Metastasis Supressor Genes	Cell invasion and metastasis	[62]
	HOTTIP	Human Fibroblast	Interaction with the WDR5/MLL complex leading in H_3_K_4_me_3_	WDR5/ MLL	HOXA locus	Gene Activation	[63]
	ANRIL	Fibroblast cell lines	H_3_K_27_me_3_ epigenetic silencing	PRC2 (SUZ12)	CDKN2A/B (p15^INK4B/A^)	Promotes cell proliferation	[64]
	LUCAT1	NSCLC	Decrease of H_3_K_27_me_3_ of target promoters through interaction with EZH2/SUZ12	EZH2/ SUZ12	p21 and p57 promoters	Cell proliferation	[65]
**Histone Acetylation**	lncPRESS1	Embryonic stem cells	Molecular decoy for SIRT6 preventing the de-acetylation of H_3_K_56_/K_9_ac marks	SIRT6	Pluripotency genes	ESCs differentiation process	[73]
**DNA methylation**	TARID	Head, neck, skin	Recruits GADD45A and TDG/BER to the TCF21 promoter leading to its activation	GADD45A	TCF21	Not well defined	[68]
**Post-Translational modification**	ANCR	Breast	Stabilizes EZH2 through regulation of ubiquitination	EZH2	EZH2	Repression of breast cancer cells migration and invasion	[69]
	LUCAT1	Esophagus	Controls DNMT1 stability by modulating ubiquitination UHRF1.	DNMT1, UHRF1	DNMT1	Impact on Esophageal Squamous Cell Carcinoma (ESCC) proliferation, migration and apoptosis	[70,71]
	MEG3	Gallbladder cancer (GBC)	Promotes EZH2 ubiquitination by increasing its phosphorylation	EZH2	LATS2, EZH2	Cell proliferation and apoptosis, Epithelial Mesenchymal Transition (EMT) progression, cell invasion	[72]
**Nuclear/Chromatin 3D architecture**	ThymoD	T-cells	Transcription of ThymoD facilitates the formation of a chromatin loop between Bcl11b promoter and Bcl11b enhancer elements	-	Bcl11b gene	Lymphoid malignancy	[74]
	Promoter of PVT1	Breast	Competes with MYC promoter for interaction with enhancers	-	MYC promoter	Cancer cell growth	[75]
	NEAT1	Paraspeckles	Differential RNA folding	-	Paraspeckles	Liver cancer	[76,77]
**Epigenetically regulated lncRNAs**	NKILA	T cells	Associates STAT1 with NF-κB signaling	NF-κΒ-IκΒα	NF-κΒ signaling process	Tumor immune evasion	[66]
	TP53TG1	Colon	YBX1 nuclear accumulation	YBX1	YBX1	Chemoresistance	[67]
